# Linear Predictive Approaches Separate Field Potentials in Animal Model of Parkinson's Disease

**DOI:** 10.3389/fnins.2020.00394

**Published:** 2020-04-24

**Authors:** Md Fahim Anjum, Joshua Haug, Stephanie L. Alberico, Soura Dasgupta, Raghuraman Mudumbai, Morgan A. Kennedy, Nandakumar S. Narayanan

**Affiliations:** ^1^Department of Electrical and Computer Engineering, The University of Iowa, Iowa City, IA, United States; ^2^DISTek Integration Inc., Cedar Falls, IA, United States; ^3^Department of Neurology, Medical School, University of Minnesota, Minneapolis, MN, United States; ^4^Shandong Provincial Key Laboratory of Computer Networks, Shandong Computer Science Center, Jinan, China; ^5^Department of Neurology, Papajohn Biomedical Institute, The University of Iowa, Iowa City, IA, United States

**Keywords:** levodopa, linear predictive coding, local field potential, mice, Parkinson's disease

## Abstract

Parkinson's disease (PD) causes impaired movement and cognition. PD can involve profound changes in cortical and subcortical brain activity as measured by electroencephalography or intracranial recordings of local field potentials (LFP). Such signals can adaptively guide deep-brain stimulation (DBS) as part of PD therapy. However, adaptive DBS requires the identification of triggers of neuronal activity dependent on real time monitoring and analysis. Current methods do not always identify PD-related signals and can entail delays. We test an alternative approach based on linear predictive coding (LPC), which fits autoregressive (AR) models to time-series data. Parameters of these AR models can be calculated by fast algorithms in real time. We compare LFPs from the striatum in an animal model of PD with dopamine depletion in the absence and presence of the dopamine precursor levodopa, which is used to treat motor symptoms of PD. We show that in dopamine-depleted mice a first order AR model characterized by a single LPC parameter obtained by LFP sampling at 1 kHz for just 1 min can distinguish between levodopa-treated and saline-treated mice and outperform current methods. This suggests that LPC may be useful in online analysis of neuronal signals to guide DBS in real time and could contribute to DBS-based treatment of PD.

## 1. Introduction

Parkinson's disease (PD) and other neurodegenerative conditions involve impaired movement and cognition. Although PD is a complex disease, a feature common to all cases is loss of midbrain dopamine neurons that project throughout the brain, including the cerebral cortex and basal ganglia (Narayanan et al., 2013). Neuronal activity as measured by electroencephalography (EEG) or local field potentials (LFPs) can be abnormal in PD patients (McCarthy et al., 2011), and aspects of these abnormalities can be recapitulated in rodent models of PD that are based on dopamine depletion (Parker et al., 2015; Alberico et al., 2017).

One effective treatment for PD is deep brain stimulation (DBS), in which high-frequency electrical current (generated by an implanted, battery-powered impulse generator) is applied directly to the subthalamic nucleus (STN) or globus pallidus to modulate PD-related neuronal activity and alleviate motor symptoms (Deuschl et al., 2006). The parameters used by clinicians performing DBS are generally adjusted only rarely—weeks or months apart. This conventional DBS (cDBS) is associated with an average battery life of 4 years with replacement requiring general anesthesia and substantial hardware costs (Bin-Mahfoodh et al., 2003).

However, recent work has suggested that ‘adaptive’ DBS has the potential to be more effective and more energy-efficient then cDBS, resulting in potentially longer battery life (Little et al., 2013, 2016a). Adaptive DBS (aDBS) refers to a deep-brain stimulation system where parameters such as stimulation voltage and frequency are changed in real time based on local brain activity. The goal of aDBS is to adjust these parameters dynamically to deliver the most effective brain stimulation. For such adjustments, current approaches of aDBS utilize the power of the beta rhythm (12–30 Hz), which can be abnormal in PD (Priori et al., 2004; López-Azcárate et al., 2010). Initial studies using this strategy have shown it to be more efficacious and efficient than non-adaptive stimulation (Little et al., 2013, 2016a; Beudel and Brown, 2016); aDBS based on the beta rhythm resulted in 27% improvement in motor scores and a 52% reduction in total electrical energy delivered (Little et al., 2013).

Although aDBS may be more effective and energy-efficient than cDBS, it faces some challenges. One of these challenges is finding an optimal control algorithm. For example, STN beta-power can be correlated with bradykinesia and rigidity but not with tremor (Meidahl et al., 2017), implying that all PD symptoms may not be ameliorated by aDBS. Other limitations of aDBS include the available computing power of the implanted system and aDBS power consumption. For example, beta-power responsive aDBS systems deliver 132 μW per side (Little et al., 2013), whereas energy consumed by a low-energy circuit for a single channel power classifier can be as low as 10μW. These constraints can impose severe limitations on using multi-dimensional feedback and more sophisticated control systems (Meidahl et al., 2017). Conventional machine-learning algorithms may require considerable processing power and processing time; however, aDBS may benefit from computationally-efficient algorithms. Moreover, aDBS requires brain stimulation to be adjusted based on ongoing neuronal activity. These “online” adjustments require that stimulation triggers be activated in real time with short latencies (in the order of milliseconds) (Little et al., 2013). Although existing algorithms can be used to determine the predictive aspects of neuronal signals (Jiang et al., 2017), in the context of adaptive applications it may be advantageous to use algorithms with as few parameters as possible. Such algorithms will confer the necessary computational efficiency and compatibility with real time processing of neuronal signals, which are inherently complex and noisy. We thus seek to develop a novel signal-processing approach that is capable of detecting features of neuronal activity in PD based on a minimal set of parameters which results in low computational latency and low device power.

We introduce an approach for analyzing neuronal activity that is based on linear predictive coding (LPC) and meets the above-described requirements (Anderson and Moore, 1979). Specifically, our approach involves rapidly encoding time-series of LFP data into a single LPC parameter. We provide a proof-of-principle demonstration that changes in this parameter can distinguish between levodopa-treated and saline-treated dopamine-depleted mice and discuss the implications for future aDBS approaches.

## 2. LPC Primer

This section provides a brief introduction to LPC, a fundamental part of our approach. For notational simplicity, vectors and matrices are denoted by bold letters throughout this section. LPC constitutes a powerful tool for predicting behaviors in time-series and for distinguishing between time-series (Anderson and Moore, 1979). Pioneering work of various groups (Makhoul, 1975; Markel and Gray, 1982; Schroeder and Atal, 1985; Atal, 2006; Schroeder et al., 2008) has made LPC the dominant technical device in speech processing, enhancement, and coding. It has also been used in EEG coding (Kiryu et al., 1994), economics (Mittnik, 1989), control theory (Gevers and Wertz, 1983), filtering (Kailath, 1968), and a host of other applications. At its core, LPC fits an autoregressive (AR) model to a time-series. Specifically, suppose one has the time-series subsequence:

(1) $$\begin{array}{r} x\left(0\right),x\left(1\right),\ldots ,x\left(L-1\right) \end{array}$$

with the arguments representing the sample indices. The *N*^*th*^ order LPC model for the time-series signal *x*(*n*) approximates it with $\hat{x}\left(n\right)$, given by

(2) $$\begin{array}{r} \hat{x}\left(n\right)=\overset{N}{\underset{k=1}{\sum }}a_{k}^{N}x\left(n-k\right) \end{array}$$

The coefficients $a_{k}^{N}$ are predictor coefficients. They are chosen to minimize the mean square error *J*, where

(3) $$\begin{array}{r} J=𝔼\left[|x\left(n\right)-\hat{x}\left(n\right){|}^{2}\right] \end{array}$$

The solution to the underlying optimization is provided by the classical Yule–Walker equations, given below (Makhoul, 1975). With complex conjugate denoted as *, we define the autocorrelation of lag *l* as:

(4) $$\begin{array}{r} r_{xx}\left(l\right)=𝔼\left[x\left(n\right)x^{*}\left(n-l\right)\right] \end{array}$$

We define the *N* × *N*, autocorrelation matrix as

(5) $$\begin{array}{r} {\text{R}}_{\text{N}}=\left[\begin{array}{cccc} r_{xx}\left(0\right) & r_{xx}^{*}\left(1\right) & \ldots & r_{xx}^{*}\left(N-1\right)\\ r_{xx}\left(1\right) & r_{xx}^{*}\left(0\right) & \ldots & r_{xx}^{*}\left(N-2\right)\\ \vdots & \vdots & \ddots & \vdots \\ r_{xx}\left(N-1\right) & r_{xx}\left(N-2\right) & \ldots & r_{xx}\left(0\right) \end{array}\right] \end{array}$$

With the vector of predictor coefficients defined as:

(6) $$\begin{array}{r} {\text{a}}^{\text{N}}=\left[\begin{array}{c} a_{1}^{N}\\ a_{2}^{N}\\ \vdots \\ a_{N}^{N} \end{array}\right] \end{array}$$

and the vector of autocorrelation functions defined as

(7) $$\begin{array}{r} {\text{r}}_{\text{N}}=\left[\begin{array}{c} r_{xx}^{*}\left(1\right)\\ r_{xx}^{*}\left(2\right)\\ \vdots \\ r_{xx}^{*}\left(N\right) \end{array}\right] \end{array}$$

The optimum **a**^**N**^ is given by

(8) $$\begin{array}{r} {\text{a}}^{\text{N}}={\text{R}}_{\text{N}}^{-1}{\text{r}}_{\text{N}} \end{array}$$

As **R**_**N**_ is Toeplitz, it is amenable to efficient inversion. Fast recursive algorithms (e.g., the Levinson-Durbin algorithm) can recursively provide **a**^**N**^ from **a**^**N−1**^. Other recursions, even for non-stationary time-series, that explicitly avoid inversion in (8) can be found in the literature (Anderson and Moore, 1979; Lopez-Valcarce et al., 2000). In this paper, we focused on first and second order LPC. The first order case is characterized by a single LPC parameter, **a**^**1**^. In the second order case, two alternative characterizations are possible. The first uses the 2-vector, **a**^**2**^. The second uses *p*_*i*_, the poles of the transfer function:

(9) $$\begin{array}{r} H\left(z\right)=\frac{1}{1-a_{1}^{2}z^{-1}-a_{2}^{2}z^{-2}}=\frac{1}{\left(1-p_{1}z^{-1}\right)\left(1-p_{2}z^{-1}\right)} \end{array}$$

Note that, for the second order case, *p*_*i*_ are the roots of the following polynomial:

(10) $$\begin{array}{r} F\left(z\right)=1-a_{1}^{2}z^{-1}-a_{2}^{2}z^{-2} \end{array}$$

Should *p*_*i*_ be a conjugate pair, which our results show that they are, their phase provides an estimate of the dominant frequency component that characterizes the time-series. Specifically, if the time-series has a dominant spectral component at frequency *f*_0_ Hz, then the LPC poles will be of the form $p_{i}=Ae^{j2\pi f_{0}T_{s}}$, where *T*_*s*_ is the sampling interval. Thus, in the second order case, poles *p*_*i*_ may provide better interpretation than the LPC coefficients.

## 3. Methods

### 3.1. Animal Experiments and Surgical Procedures

Eight male C57/B6 wild-type mice (Harlan, Madison, WI) were included in this study. Details of animal behavior and experimental protocols were described in depth previously (Alberico et al., 2017). Some data from Alberico et al. (2017) were used for our present study. Note that this paper describes a proof-of-principle rather than a hypothesis-testing study.

All procedures were approved by the Animal Care and Use Committee at The University of Iowa. We used a standard rodent model of PD, in which the median forebrain bundle (MFB) is depleted of dopamine by unilateral injection of the neurotoxin 6-OHDA (Dauer and Przedborski, 2003; Schober, 2004) using stereotaxic procedures. This dopamine-depletion procedure causes cell death of dopamine neurons in the injected hemisphere and models various aspects of PD including movements, electrophysiology (an increase in 13–30 Hz power), and response to treatments like levodopa and DBS (Deumens et al., 2002; De Jesús-Cortés et al., 2015; Parker et al., 2015; Alberico et al., 2017; Kim et al., 2017).

**Figure 1:**
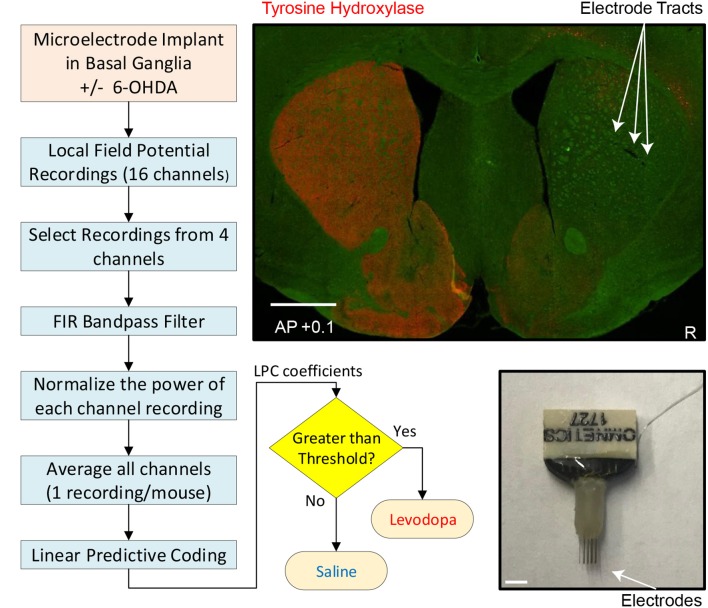
Experimental and analytic approach. **Left** and **bottom middle**: Flow chart depicting individual steps from implantation of electrodes to readout of whether mouse had been treated with levodopa or saline (sham-treated control). **Top right**: coronal section of striatum (0.1 mm anterior to bregma) from mouse subjected to dopamine depletion on right side (injection with neurotoxin 6-OHDA) to model PD and immunostained for tyrosine-hydroxylase (red), the rate limiting enzyme for dopamine synthesis in dopaminergic axons. Sites of microelectrode-array implantation are indicated by arrows. White scale bar is 1 mm. **Bottom right**: microelectrode.

Mice were anesthetized using ketamine (100 *mg*/*kg*) and xylazine (10 *mg*/*kg*) and injected with desipramine (25 *mg*/*kg*; i.p.) to protect catecholaminergic neurons other than the dopaminergic subset. Four animals (mice 1–4) were subjected to unilateral injection of 1μ*L* of 6-OHDA (1μ*g*/μ*L*; dissolved in 0.02–0.03% ascorbic acid; example of dopamine-depletion shown in Figure 1) into the MFB (from bregma: AP: −1.2, ML: −1.2, DV: −4.7 from dura). Four different animals (mice 5–8) were stereotactically injected with 0.02% ascorbic acid at the same site to control for effects of dopamine-depletion. During these surgeries, one 16-channel stainless-steel microwire array was lowered into the dorsal striatum (Figure 1; 50μ*m*, 4 × 4 with contacts 250μ*m* apart. MicroProbes, Gaithersburg, MD; 8 animals: AP: +0.1, ML: 2.0, DV: −3.0). In all animals, the arrays were grounded via a stainless steel wire wrapped around two skull screws. The craniotomy was sealed with cyanoacrylate (“SloZap,” Pacer Technologies, Rancho Cucamonga, CA) and methyl methacrylate (dental cement; AM Systems, Port Angeles, WA).

After a week of recovery, MFB-injected animals were screened for effectiveness of unilateral dopamine depletion using the amphetamine-induced rotation test. Animals were injected with amphetamine (5 *mg*/*kg*; i.p.) to induce ipsilateral rotation, and performance was recorded 30 min post injection (Healy-Stoffel et al., 2012; Chotibut et al., 2014). A criterion of 6 ipsilateral amphetamine-induced rotations per minute was used to verify dopamine-depletion; all mice met this criterion (Paquette et al., 2009).

### 3.2. LFP Recordings

After assessing all mice as suitable for analysis, a multi-electrode recording system (Plexon, Dallas, TX) was used to record activity in neuronal ensembles. Electrical activity of LFP channels was recorded in parallel with single unit channels using a wideband board. During signal acquisition, LFPs were sampled at 1,000 Hz, high pass filtered at 0.05 Hz and low pass filtered at 200 Hz (Plexon). LFPs were recorded from the dorsal striatum of both dopamine-depleted mice (6-OHDA injected: mice 1–4) and control mice (ascorbic-acid injected: mice 5–8), both following injection with saline and following injection with levodopa, a dopamine precursor used to treat motor symptoms of both dopamine-depleted mice. For levodopa sessions, we administered 20 *mg*/*kg* of L-DOPA-methylester (levodopa; Sigma) dissolved in 0.09% NaCl sterile saline at a concentration of 4 *mg*/*mL* with Benserazide (2 *mg*/*mL*; Sigma).

The LFPs were recorded from the dorsal striatum during a 40-min period of free movement after injection. During recording sessions, animals were placed in a 6-inch diameter, 7-inch tall plexiglass chamber. Animals were awake and mobile for the entire session as verified by video camera and 3-dimensional motion capture, which was performed according to methods described in detail previously (Alberico et al., 2017). Briefly, OptiTrack Prime infrared cameras tracked the position of infrared reflective spheres attached to the animals' headstage sampled at 120 frames/s. Levodopa-related activity was verified by movement velocity and experimenter observation of the animal. All animals were awake and freely-moving as monitored by an experimenter throughout the recording. Movements were quantified via infrared tracking of head position (OptiTrack). Beyond levodopa vs. saline administration, all other procedures were identical. The same animals were injected on different days with levodopa and saline.

For all mice, neuronal activity was recorded from microelectrodes implanted into the striatum. For all recordings levodopa or saline was injected immediately prior to each recording session. LFPs were sampled at 1 kHz and recorded from the same 4 of 16 channels (#1, 5, 9, and 13) in all mice.

### 3.3. Post-recording Procedures

After the mouse experiments were completed, the animals were euthanized by injection of 100 *mg*/*kg* sodium pentobarbital and then transcardially perfused with 4% paraformaldehyde. Brains were post-fixed in 4% paraformaldehyde and cryoprotected in 30% sucrose and then sectioned using a cryostat. Brain slices were mounted and stained for tyrosine hydroxylase (TH; polyclonal rabbit anti-TH, 1:500) (Millipore, Temecula, CA). Sections were imaged using an Olympus Systems VS120 microscope (Figure 1).

### 3.4. Signal Processing

Our goal was to obtain one LFP time-series from each mouse in order to compute LPC coefficients. The steps of our signal processing method are illustrated with a flowchart in Figure 1 and described below.

#### 3.4.1. Filtering

For each mouse, LFP recordings of 4 channels were obtained and data from each channel was filtered separately using a zero-phase, Finite Impulse Response (FIR) 2.5–50 Hz bandpass filter. No other artifact removal algorithm was used on the signals.

#### 3.4.2. Normalization

Filtered data were then normalized to retain unit power. Specifically, for a time-series defined in (1), signal power is given by,

(11) $$\begin{array}{r} P=\frac{1}{L}\overset{L-1}{\underset{n=0}{\sum }}|x\left(n\right){|}^{2} \end{array}$$

Then, the normalized time-series will be,

(12) $$\begin{array}{r} \frac{x\left(0\right)}{\sqrt{P}},\frac{x\left(1\right)}{\sqrt{P}},\ldots ,\frac{x\left(L-1\right)}{\sqrt{P}} \end{array}$$

#### 3.4.3. Averaging

Normalized LFP recordings from 4 channels were then averaged in the time domain to obtain one single time-series for each mouse.

### 3.5. Feature Extraction Using LPC

After processing the LFP data, we used first and second order LPC for feature extraction. For first order LPC, a single LPC parameter, *a*^1^ was computed using (8). For the second order LPC, poles *p*_*i*_ were calculated using (10).

For the striatal LFP data from healthy control mice (no dopamine depletion, mice 5–8), first order LPC coefficients are calculated for the entire 40-min session. We investigated whether the session could be characterized by a value that consistently marks the separation between the first order LPC parameters for levodopa-injected vs. saline-injected treatments in dopamine-depleted animals.

For the striatal LFP data from four dopamine-depleted mice (mice 1–4), we calculated LPC coefficients for three cases.

#### 3.5.1. Full-Length Data

First, we computed the first order LPC coefficients for the entire 40-min trial for the striatal LFP data from 4 dopamine-depleted mice (mice 1–4) and observed the performance. Then, the second order LPC poles, *p*_*i*_ were computed for the entire sessions.

#### 3.5.2. Segmented Data

Next, we divided the LFP data for each 40-min trial into epochs of 1, 3, 10, and 40 min (a full-length window) and obtained first order LPC coefficients for each data segment to observe the stability and the amount of separation for different signal lengths. In order to determine the effect of movements on the LPC coefficients, we investigated whether LPC coefficients were related to movement velocity. For each saline and levodopa session, we used linear mixed-effects model for LPC coefficients with fixed effects for average velocity and random effects for intercept and average velocity grouped by the mouse number. This was done with 1 and 3 min epochs.

#### 3.5.3. Expanding Time-Window

To emulate the performance of LPC coefficients for real time data, we next calculated the LPC coefficients using an expanding time window. An initial estimate of an LPC parameter can be made based on a small number of samples and it can be updated in real time as each additional sample is measured (Lopez-Valcarce et al., 2000). We used a 1 kHz sampling rate; thus, new samples are available every millisecond and once the initial LPC estimate is made, updates can be generated within a few milliseconds. The first order LPC coefficient was generated for the first second of LFP data. The window was then expanded in increments of 1 s.

### 3.6. Performance Comparison

We compared our approach with several established methods from the literature (López-Azcárate et al., 2010; de Hemptinne et al., 2013; Sanders et al., 2013; Little et al., 2016a,b). The goal was to observe how well our method separates saline and levodopa sessions for a PD mouse model compared to state-of-the-art methods. Striatal LFPs from dopamine-depleted PD mice were used for this purpose. Analyzing power spectral density (PSD) and Phase-amplitude coupling (PAC) are two common approaches to analyze LFP data. We compared our approach with methods based on these two techniques (López-Azcárate et al., 2010; de Hemptinne et al., 2013; Sanders et al., 2013; Little et al., 2016a,b).

For PSD-based approaches, we focused on beta-power (12–30 Hz), which is currently used as a threshold trigger for aDBS and can characterize ON vs. OFF Parkinsonian motor state (López-Azcárate et al., 2010; Little et al., 2016a,b). We computed the beta-power using Little et al. (2016a,b). For PAC-based approaches, modulation of the amplitude of oscillations in high frequency band (HFO) caused by the phase of low frequency band is measured (López-Azcárate et al., 2010; Tort et al., 2010; de Hemptinne et al., 2013; Sanders et al., 2013). We measured PAC according to the method of de Hemptinne et al. (2013). —coupling between β-phase (13–30 Hz) and γ- amplitude (50–200 Hz), and the method described by López-Azcárate et al. (2010) coupling between low-beta (12–30 Hz) and HFO (200–350 Hz). We measured 36 PAC modulation indices by using six spectral sub-bands within the 3–60 Hz range (delta: 3–4 Hz, theta: 5–7 Hz, alpha: 8–11 Hz, low beta: 12–19 Hz, high beta: 20–30 Hz, and gamma: 31–60 Hz) and computed weighted combinations of these modulation indices by using the method of canonical correlation as described by Sanders et al. (2013).

We tested these methods with striatal data from dopamine-depleted PD mice. First, entire 40-min sessions were used and then we segmented the data by 3-min epochs. For an entire 40-min session, only one LFP signal can be analyzed while in the case of 3-min epochs, 13 signals can be analyzed using 13 epochs from the entire LFP signal. For the method described by Sanders et al. (2013), notice that the total number of signals must be greater than the number of PAC measurements so the method can only be applied on the 3-min epochs.

We also compared the computational efficiency of our proposed method with the aforementioned methods by calculating the total computation time. We used 3-min epochs for this purpose. We ignored all filtering processes while calculating the computation time for a fair comparison of the methods. The calculation of time and execution of these methods was done in Matlab (version: R2019a) with Intel(R) Core(TM) i7-8750H CPU @ 2.2 GHz machine.

### 3.7. Statistical Analysis

Due to the small dataset, two non-parametric methods were chosen for statistical analysis: Wilcoxon rank sum test and Kruskal–Wallis test. First order LPC coefficients obtained from both the 40-min sessions and the 3-min epochs of striatal data from the dopamine-depleted mice were used for the tests. We also used these tests to analyze statistical significance of beta-power and phase-amplitude coupling changes between saline and levodopa-treated sessions for both 40- and 3-min epochs using the PAC-based (López-Azcárate et al., 2010; de Hemptinne et al., 2013; Sanders et al., 2013) and PSD-based (Little et al., 2016a,b) approaches.

## 4. Results

This section has three parts. The first two are on the analysis of striatal data from dopamine-depleted PD mice and healthy control mice, respectively. In the last part, we compared the performance of our approach with established methods and presented the statistical analysis of our findings.

### 4.1. Analysis of Striatal LFP From Dopamine-Depleted PD Mice

**Figure 2:**
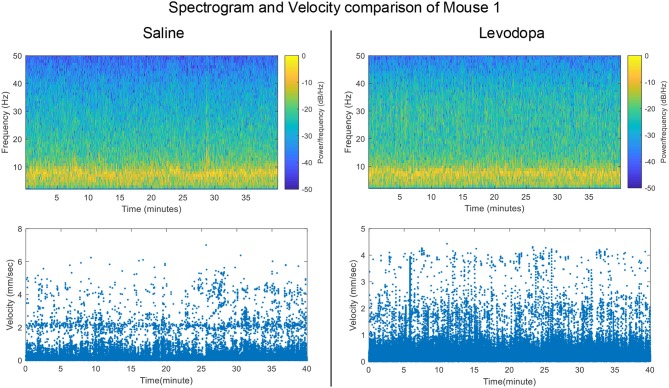
Spectrogram of Striatal LFP signals and velocity comparisons in saline-injected session **(left)** and levodopa-injected session **(right)** of Mouse 1.

Striatal LFP data from 4 dopamine-depleted mice (mice 1-4) were analyzed. Each mouse had LFP data for two sessions: saline and levodopa. Of note, dopamine-depleted mice moved with less average velocity in saline-injected sessions (0.05 ± 0.01 mm/s; mean ± SEM) compared with when they were given levodopa [0.12 ± 0.01; paired *t*_(3)_ = 6.2, *p* < 0.01]. Figure 2 shows the velocity and spectrogram comparison of 40 min sessions of saline and levodopa from Mouse 1.

#### 4.1.1. LPC Coefficients for Full-Length Data

**Figure 3:**
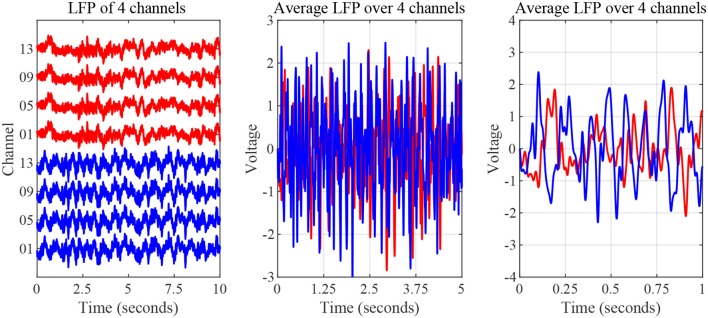
Striatal LFP signals from saline-injected (red) and levodopa-injected session of dopamine-depleted mouse 1 (blue). **Left**: Signals as measured by microelectrode array (raw signal) of 4 channels. **Middle**: Average signal over 5-s period. **Right**: Average signal over 1-s period.

**Figure 4:**
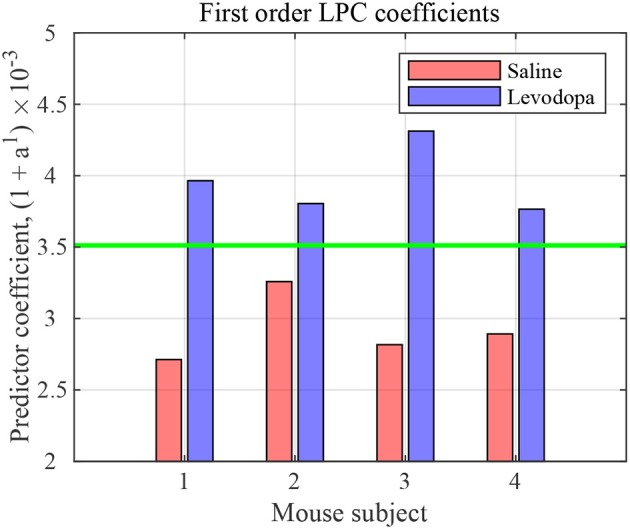
First order LPC coefficients for striatal LFPs in saline-injected (red) and levodopa-injected (blue) dopamine-depleted mice. These coefficients are sufficient for binary classification of the LFP data for mice 1–4. The threshold (green) is calculated by taking the average of the minimum value in levodopa session and the maximum value in saline session.

The nature of time-domain LFP data are depicted in Figure 3 using saline and levodopa sessions from dopamine-depleted mouse 1. We investigated whether the entire 40-min session could be characterized by a value that consistently separates the first order LPC parameters of levodopa-treated session vs. control (saline-treated) session of dopamine-depleted mice. Figure 4 shows that such a boundary exists for all four mice, suggesting that the onset of signals reflective of dopamine depletion in the PD mouse model is quick and efficient.

**Figure 5:**
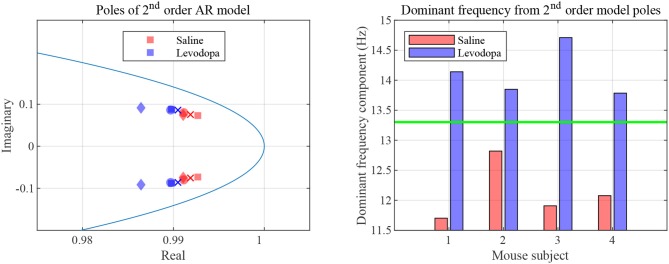
Second order LPC model with dopamine-depleted mice (mice 1–4). **Left:** second order poles for striatal LFPs in dopamine-depleted mice. Shown are the complex conjugate pairs *p*_1_ and *p*_2_, for data sets for dopamine-depleted mice following treatment with saline (red) or levodopa (blue). The unit circle line is shown in light blue. The *X* and *Y* axes represent the real and imaginary, respectively, parts of the poles. Each symbol represents a single pole from a single epoch. Squares represent data from mouse 1, circles from mouse 2, diamonds from mouse 3, and crosses from mouse 4. **Right:** dominant frequency components obtained from the second order poles. The threshold (green) is obtained by averaging the maximum frequency for saline and minimum frequency for levodopa session.

**Figure 6:**
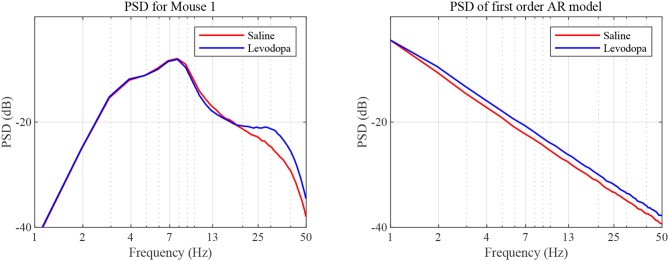
PSD as a function of frequency. PSD was determined based on sampled LFP **(left)** and our first order AR model **(right)**, for data sets generated for dopamine-depleted Mouse 1 following treatment with saline (red) or levodopa (blue).

In the case of second order LPC coefficients for these 40-min sessions, the outcome was similar. Figure 5 depicts the poles, *p*_*i*_, in (9) and the dominant frequencies for each mouse and demonstrates the differences between saline and levodopa sessions of the dopamine-depleted mice. The separation of the second order LPC coefficients did not improve over their first order counterparts. The poles of the second order LPC are complex conjugate pairs. Their phase equals the dominant frequency normalized by the sampling rate. Figure 5 shows that the dominant frequencies of levodopa sessions are in the range of 13.8 Hz to 14.7 Hz which corresponds to the beta band (12–30 Hz) while the frequencies of the saline sessions ranged from 11.7 to 12.8 Hz which are in the lower end of the same band. Nonetheless, the separation enhancement between saline and levodopa session is marginal in going from first to second order, and is overwhelmed by the fact that first order LPC computations are twice as fast as second order LPC. Figure 5 also shows that the dominant frequency is lower under saline-injected conditions in a dopamine-depleted mouse than following levodopa injection, consistent with the power spectral density (PSD) of the mouse in the levodopa-treated vs. saline-treated states (Figure 6). We also considered the PSD of the first order AR model provided by the optimum predictor and found a similar result which is illustrated in Figure 6.

Both analyses indicate that the LFP is of smaller bandwidth with saline-injections compared to levodopa. In other words, levodopa increases the bandwidth of striatal LFPs in dopamine-depleted animals which is an essential attribute captured by both first and second order LPC for separating levodopa and saline sessions. The lack of discernible separability in second order LPC over first order also indicates that the contribution of additional features by higher order LPC in separating levodopa and saline sessions, if any, is marginal and does not justify the additional computations second order LPC requires.

#### 4.1.2. LPC Coefficients for Segmented Data

**Figure 7:**
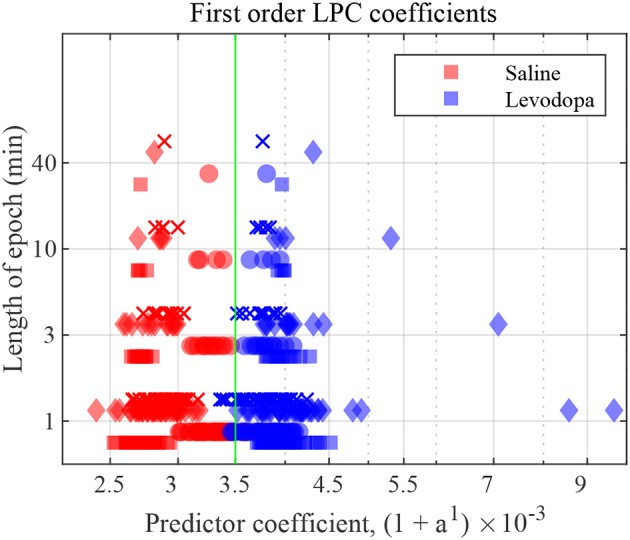
First order LFP coefficients for dopamine-depleted mice following injection with saline (red) or levodopa (blue). Entire 40-min data set was divided into epochs of lengths 1, 3, 10 and 40 min. *Y* axis represents length of each epoch, and *X* axis the value of the coefficient *a*^1^ plotted as (1 + *a*^1^) × 10^−3^. Each symbol represents a single coefficient from a single epoch. Squares are data from mouse 1, circles mouse 2, diamonds mouse 3, and crosses mouse 4. The threshold (green) is calculated by taking the average of the minimum value in levodopa session and the maximum value in saline session for 40 min epochs.

Figure 7 depicts the first order LPC coefficients obtained for all four mice for different epoch lengths. For all mice, the separation between the LPC parameters before and after treatment is clear when epochs are 3 min or longer in duration. In mice 1 and 3 this separation is evident even for 1-min epochs. These patterns are consistent amongst mice 1-4, the difference being that some show a clearer separation for the saline-injected vs. levodopa data sets.

**Figure 8:**
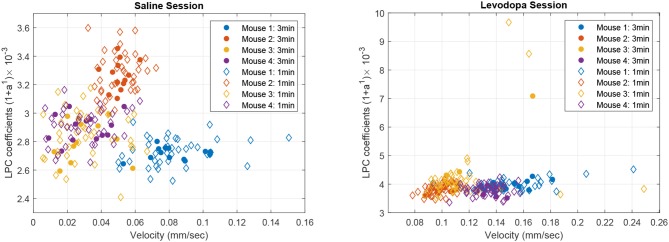
Scatter plot of first order LPC coefficients and average velocity for 3 min (solid dots) and 1 min (diamonds) epochs of striatal LFP signals in dopamine-depleted mice (mice 1–4) following treatment with saline **(left)** or levodopa **(right)**.

Figure 8 shows the correlation between velocity and LPC coefficients with scatterplots of the first order LPC coefficients compared with average movement velocity for 1 and 3 min epochs. Table 1 provides the details of the linear fixed-effects models for LPC coefficients with fixed effects for average velocity using 1 and 3 min epochs of saline and levodopa session. In both cases of 1 and 3 min epochs, large confidence interval along with high standard error and large *p*-value (>0.05) of the estimated coefficient for average velocity indicated the lack of statistically significant effects of average velocity on the LPC coefficient.

**Table 1:** Summary of the linear mixed-effects models of the average velocity and the LPC coefficient of mice 1–4 in saline (left) and levodpoa (right) session with 3-min (upper half) and 1-min (lower half) epochs.

	**Saline session**				**Levodopa session**				
	Dependent variable: LPC coefficient				Dependent variable: LPC coefficient				
	Coeff. Est. (95% CI)	Standard error	*t*-score	*p*-value	Coeff. Est. (95% CI)	Standard error	*t*-score	*p*-value	
Velocity	1.67 (−1.2 to 4.5)	1.42	1.17	0.25	11.3 (−4.1 to 26.7)	7.66	1.47	0.15	3 min
Const.	2.86 (2.7 to 3)	0.08	36.48	1.1e-37	2.69 (1.1 to 4.3)	0.79	3.42	1.3e-3	
Velocity	1.38 (−0.2 to 2.9)	0.8	1.72	0.09	5.3 (−0.6 to 11.2)	2.98	1.77	0.08	1 min
Const.	2.87 (2.7 to 3)	0.09	32.8	2.9e-72	3.34 (2.7 to 3.9)	0.3	11.13	1.3e-21	

*95% CI, 95% Confidence interval; Const., Constant interception; Coeff. Est., Coefficient Estimate; t-score and p-value shows the t-statistics for testing the null hypothesis that the coefficient is equal to zero*.

#### 4.1.3. LPC Coefficients for Expanding Time-Window

**Figure 9:**
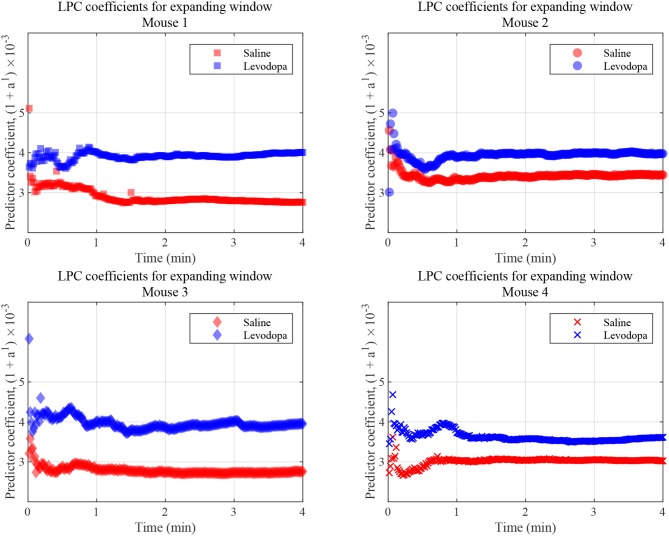
LFP coefficients with window size expanding over time, for the indicated dopamine-depleted mouse. LPC coefficients were calculated using window that expands in increments of 1 s for a 4-min time course. Coefficients for dopamine-depleted mice (mice 1–4) following treatment with saline (red) or levodopa (blue). Each symbol represents a single coefficient from a single window that includes data for all the LFP samples up until that time point.

LPC coefficients were calculated for expanding time windows and the resulting coefficients are shown for the first 4 min of each session in Figure 9 which illustrates that, in this context, the coefficients for Mouse 3 converged almost immediately and those for the other mice converge within 1 min. These results are consistent with those shown in Figure 7, in which segmented data were used. Thus, levodopa and saline sessions of these four dopamine-depleted mice showed clear separation with a single LPC parameter calculated and updated based on an expanding window and less than a minute's worth of LFP data.

### 4.2. Analysis of Striatal LFP From Healthy Control (Non-PD) Mice

**Figure 10:**
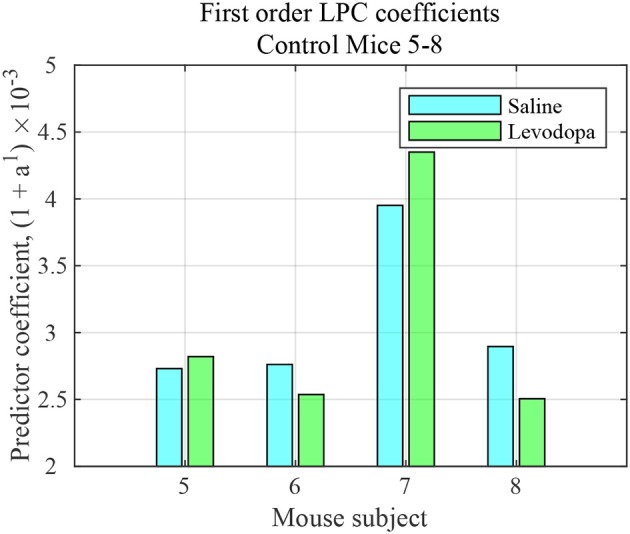
First order LFP coefficients for striatal signals in mice with intact dopamine (healthy control) mice (mice 5–8) following treatment with saline (light blue) or levodopa (green).

The striatal LFP data from healthy control (i.e., mice without dopamine depletion: Mice 5-8) were analyzed where each mouse had saline and levodopa session. Figure 10 shows a comparison of the LPC coefficients for control mice following administration of levodopa vs. saline. In this group of mice, the signals under the two conditions could not be distinguished using first order LPC. These results are consistent with our findings that the dopamine precursor levodopa has minimal effects in mice with intact dopamine, likely because excess levodopa is eliminated through normal homeostatic mechanisms (Alberico et al., 2017).

### 4.3. Performance Comparison

**Figure 11:**
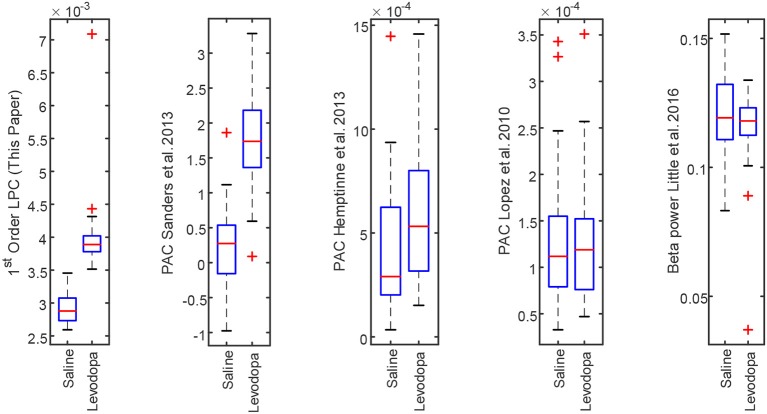
Box plot comparison of results obtained using striatum LFP data with 3-min epochs for saline vs levodopa session of dopamine-depleted mice (mice 1–4). From left: LPC based approach, PAC method by Sanders et al. (2013), de Hemptinne et al. (2013), López-Azcárate et al. (2010), and Beta power-based method by Little et al. (2016a,b).

The LPC based approach was compared with the established methods for striatal LFPs from dopamine-depleted PD mice. Figure 11 compares each method for segmented data with 3-min epochs. Table 2 provides the summary of the statistical analysis for 3- and 40-min data. Differences were quantified by using the Wilcoxon rank-sum test and the Kruskal–Wallis test from 52 signals per condition for 3-min data and 4 signals per condition for 40-min data, which demonstrates that only LPC based approach achieved statistical significance in saline vs levodopa separation with *p* < 0.05 for 3- and 40-min data, although the PAC method by Sanders et al. (2013) and de Hemptinne et al. (2013) showed statistical significance for 3-min epochs. In 3-min data, the method by Sanders et al. (2013) showed promising results but it has some limitations. Specifically, weights for the calculation of composite modulation indices, which are used as the final outcome, are determined by analyzing the whole dataset of signals which are computationally expensive and heavily data-dependent as the weights will vary for a new set of data. This increases the complexity of the execution and reduces the generality of this method. Taken together, these data demonstrate that LPC provides reliable separation compared to other methods independent of epoch length.

**Table 2:** Summary of statistical analysis of the outcomes from first order LPC and other established approaches with 40 min (left) and 3 min (right) epochs.

**Method**	**40-min epoch**		**3-min epoch**	
	**Wilcoxon**	**Kruskal–Wallis**	**Wilcoxon**	**Kruskal–Wallis**
				
First Order LPC approach	0.0286*	0.0209*	1.54e-18*	1.5e-18*
PAC: (Sanders et al., 2013)	-	-	5.62e-16*	5.47e-16*
PAC: (de Hemptinne et al., 2013)	0.6857	0.5637	0.006*	0.006*
PAC: (López-Azcárate et al., 2010)	0.8857	0.7728	0.6941	0.6917
Beta Power: (Little et al., 2016a,b)	0.6857	0.5637	0.2459	0.2445

Statistical significance requires p-values < 0.05. Such p-values are marked with *.

**Figure 12:**
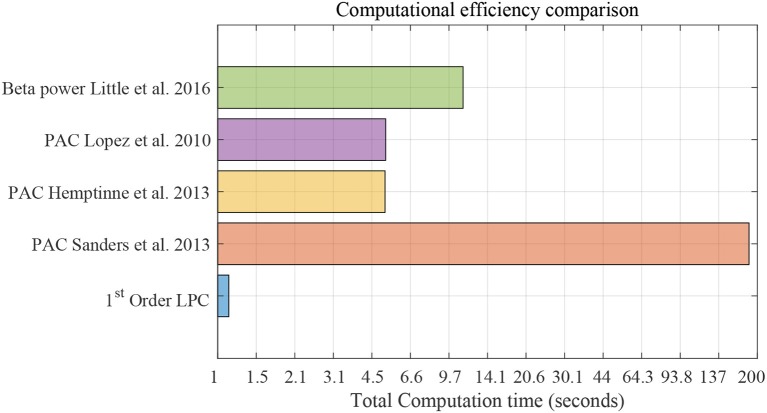
Computational efficiency comparison using striatum LFP data with 3-min epochs for saline vs levodopa session of dopamine-depleted mice (mice 1–4). *X*-axis is total computation time in seconds on log scale. Compared methods are: LPC based approach, PAC method by Sanders et al. (2013), de Hemptinne et al. (2013), López-Azcárate et al. (2010), and Beta power-based method by Little et al. (2016a,b).

Figure 12 shows the computational efficiency of the LPC based approach and the above established methods for 3-min data. Total computation time for the LPC based approach was 1.1 s. de Hemptinne et al. (2013) had the lowest computation time (5.17 s) among the established methods. The results show that LPC based approach is almost 5 times faster than the fastest established method. It should be mentioned that the LPC based approach can be executed in a recursive fashion after the first estimation which was not implemented here. This can make the approach even more efficient and amenable to real time applications.

## 5. Discussion and Conclusion

Our intent in this study is not to describe LFPs in animal models of PD, whose behavior and neurophysiology has been well-established in prior work (Betarbet et al., 2002; Alberico et al., 2017). Rather, our goal is to describe a proof-of-principle of the LPC approach. Our study indicates that first and second order LPC coefficients distinguish signals from a mouse model of PD after treating the animals with either saline or levodopa. Our data suggest that a single LPC parameter is enough to classify, that it can be updated every second, and that only one update is required. Given the short durations required (as little as 1 min), the amenability of LPC for rapid online implementation, and the extremely small number of predictive coefficients needed, an LPC-based method seems well-suited for use as a trigger mechanism for adaptive brain stimulation. We have analyzed striatal LFP data from healthy mice treated with either saline or levodopa. For healthy mice, the signals in levodopa- vs. saline-treated sessions cannot be distinguished by first order LPC. Thus, the method is not distinguishing between the mere presence or absence of levodopa treatment. Rather it is detecting the benefits of levodopa only in dopamine-depleted mice. Although this study uses channel-averaged signals from each mouse, the approach can be extended to include multichannel analysis and be potentially applicable to human EEG data for the detection of cortical signals in PD.

Although signal-processing tools such as AR models, Kalman filtering and nonlinear neural network-based models have been used in previous studies of neural signals (Pfurtscheller et al., 1998; Coyle et al., 2005; Sung-Phil et al., 2008; Coyle, 2009), our results are particularly intriguing in that simple first order LPC is sufficient for making the desired distinction. Current spectral approaches based on Fourier transforms or non-linear cross-frequency approaches generally require measurement of a larger number of parameters and are much more computationally intensive. Thus, they are more challenging to integrate into real time applications as they reduce battery life. The fact that a simple first order LPC model is capable of such a distinction is remarkable, as no studies to date have reported a first order LPC-model capable of distinguishing between manifestly non-linear phenomena. Finally, although LPC is sufficient for detecting differences caused by the injection of levodopa in dopamine-depleted mice, it does not distinguish between the administration of levodopa and saline in animals without dopamine depletion. These data provide a proof-of-principle demonstration that a first order LPC may useful for indexing the disease state in humans with PD. Specifically, aDBS may calculate LPC coefficients from basal ganglia LFPs, and use LPC parameters as a control signal. If the LPC parameters are abnormal, aDBS could theoretically be adjusted to bring the LPC parameters closer to the range of levodopa-treated and/or healthy humans, although determining these thresholds will require considerable clinical efforts.

These results are of interest because they are able to separate striatal LFPs from dopamine-depleted animals with saline- and levodopa-treatments based on a single parameter and a few minutes of data. While there is little doubt that today's machine-learning algorithms can reliably achieve such separation, first order LPC's simplicity makes it uniquely compatible with the computational power and energy efficiency required by aDBS applications. This method is a significant advance toward our goal of developing novel signal-processing approaches that are compatible with real time triggers of brain stimulation in PD models. The experiments reported here involve LFP data sampled at 1 kHz. In principle, it is possible to sample at rates as high as 40 kHz. Given that at 1 kHz sampling yields only one data point per millisecond, a faster sampling rate yielding more data may lead to faster convergence. However, we note that we are sampling a biological phenomenon of medication and pathological state and the necessary effects may not manifest over a shorter duration. Still, LPC may be helpful in the design of adaptive and responsive brain stimulation systems where a rapid and robust LPC-based analytical framework might enable more rapid and reliable convergence to effective stimulation parameters or more effective guidance of surgical approaches (Beudel and Brown, 2016; Telkes et al., 2016).

Our work is limited to a proof-of-principle demonstration in a rodent model; further studies will be required to correlate LPC parameters with the effects of dopamine depletion on motor and non-motor function in rodents. Furthermore, we are studying LFPs, and it is possible that further detail is available from single unit neuronal recordings, although this is not immediately applicable to human aDBS. In addition, because levodopa increases movement in dopamine-depleted mice we cannot be certain if differences in striatal LFP captured by LPC are a cause or a consequence of motor changes, although there is considerable data that neurons in this striatal region play a causal role in modulating movement (Kravitz et al., 2010). Regardless of whether LFP differences captured by LPC are the cause or consequence of movement, LPC-based analyses may be useful for aDBS as they can reflect the dopaminergic state of the striatum, and function as an effective trigger to optimize and adjust aDBS. Of note, dopamine-depleted mice treated with levodopa can result in levodopa-induced dyskinesias (LIDs; Fasano et al., 2010; Alberico et al., 2017). Our LPC metrics may be affected by LIDs. Future studies might develop LPC metrics to differentiate LIDs or other aspects of striatal LFPs.

This demonstration is a key step as understanding the human effects of dopamine-depletion in humans is not straightforward. Whereas it is possible to record LFPs from some structures within the human basal ganglia, recording from the specific brain regions that are directly affected by dopamine, such as the striatum, remains a major challenge. Thus, future recordings in rodent models, as well as humans, will be essential for the development and refinement of novel therapeutic applications for PD patients. It is possible that the LPC values reflect distinct brain states between dopamine-depleted mice treated with saline, those treated with levodopa, and healthy controls. We note that our aim is not to describe these brain states which could be related to complex features of dopamine signaling in the striatum, but to describe a proof-of-principle demonstration of a novel signal processing algorithm in which a single LPC parameter can be used to distinguish perturbations in striatal LFPs as a function of dopaminergic manipulations.

PD is characterized by abnormal beta-oscillations in the range of 12–30 Hz within the basal ganglia (Soikkeli et al., 1991; Priori et al., 2004; Mallet et al., 2008; López-Azcárate et al., 2010). Previous studies of PD patients have suggested that power may shift in the beta-band (Priori et al., 2004) and that this shift can be modulated by activity (Brown et al., 2001; Cassidy et al., 2002; Özkurt et al., 2011). Moreover, the power of these oscillations can be modulated by levodopa, a change that could potentially be useful for DBS applications (Giannicola et al., 2010; Whitmer et al., 2012; Beudel and Brown, 2016). These approaches analyze the envelope of beta-power and thus require a temporal window adequate for Fourier/wavelet-based analysis (Little et al., 2013, 2016a). The LPC coefficients reflect more than just changes in the beta band (12–30 Hz) power and take a more holistic view of spectral changes. Because LPC analyses are first order readouts, they can capture signal characteristics of PD using relatively few parameters. Thus, they have the potential to be more rapidly and readily configurable than current spectral methods. Extensive future work with animal models and PD patients will help refine LPC applications for diagnosing and treating human disease. Of note, human PD is a multifaceted disease that is only partially captured by animal models. Some features of human PD, such as resting tremor and freezing-of-gait, are not well-modeled in rodents. Thus, our work will have to be extended to primate or human models to explore if LPC can capture the range of PD sympatomatology.

## Data Availability Statement

The raw data supporting the conclusions of this article will be made available on request to the corresponding author, without undue reservation, to any qualified researcher.

## Ethics Statement

The animal study was reviewed and approved by Animal Care and Use Committee at The University of Iowa.

## Author Contributions

SD, RM, and NN conceived and designed the study. MA, SD, and JH performed the research. SA, MK, and NN collected and assembled the data. MA, SD, and RM analyzed and interpreted the data. MA wrote the manuscript. All authors approved the final version of the manuscript.

## Conflict of Interest

JH was employed by the company DISTek Integration Inc., Cedar Falls, IA, USA. The remaining authors declare that the research was conducted in the absence of any commercial or financial relationships that could be construed as a potential conflict of interest.
